# SPI1-mediated macrophage polarization aggravates age-related macular degeneration

**DOI:** 10.3389/fimmu.2024.1421012

**Published:** 2024-06-24

**Authors:** Siyi Qi, Yihan Zhang, Lingjie Kong, Daode Bi, Hongyu Kong, Shujie Zhang, Chen Zhao

**Affiliations:** ^1^ Eye Institute and Department of Ophthalmology, Eye & ENT Hospital, Fudan University, Shanghai, China; ^2^ Key laboratory of Myopia and Related Eye Diseases, NHC, Key laboratory of Myopia and Related Eye Diseases, Chinese Academy of Medical Sciences, Shanghai, China; ^3^ Shanghai Key Laboratory of Visual Impairment and Restoration, Shanghai, China; ^4^ Department of Neurology and Geriatrics, Fujian Institute of Geriatrics, Fujian Medical University Union Hospital, Fuzhou, Fujian, China

**Keywords:** SPI1, transcription factor, AMD, CNV, macrophage polarization, innate immunity

## Abstract

**Objective:**

This study revealed a core regulator and common upstream mechanisms for the multifaceted pathological processes of age-related macular degeneration (AMD) and provided proof-of-concept for this new therapeutic target.

**Methods:**

Comprehensive gene expression analysis was performed using RNA sequencing of eye cup from old mice as well as laser-induced choroidal neovascularization (CNV) mouse model. Through integrative analysis and protein-protein interaction (PPI) analysis, common pathways and key transcription factor was identified simultaneously engaged in age-related retinal degeneration and CNV, the two typical pathological process of AMD. Subsequently, the expression changes of *Spi1*, the key regulator, as well as the alternation of the downstream mechanisms were validated in both models through qRT-PCR, Elisa, flow cytometry and immunofluorescence. Further, we assessed the impact of *Spi1* knockdown *in vitro* and *in vivo* using gene intervention vectors carried by adeno-associated virus or lentivirus to test its potential as a therapeutic target.

**Results:**

Compared to corresponding controls, we found 1,939 and 1,319 genes differentially expressed in eye cups of old and CNV mice respectively. The integrative analysis identified a total of 275 overlapping DEGs, of which 150 genes were co-upregulated. PPI analysis verified a central transcription factor, SPI1. The significant upregulation of *Spi1* expression was then validated in both models, accompanied by macrophage polarization towards the M1 phenotype. Finally, SPI1 suppression significantly inhibited M1 polarization of BMDMs and attenuated neovascularization in CNV mice.

**Conclusion:**

This study demonstrates that SPI1 exerts a pivotal role in AMD by regulation of macrophage polarization and innate immune response, offering promise as an innovative target for treating AMD.

## Introduction

AMD as a degenerative eye condition that mainly impacts the elderly, is a major contributor to worldwide vision impairment (1–3). This condition arises when the macula gets damaged due to the process of aging. AMD is expected to influence approximately 288 million individuals worldwide by 2040 (4). The precise reason behind AMD remains incompletely comprehended. It is generally recognized to arise from a combination of genetic and environmental elements, with advancing age being the determinant. Risk factors identified so far include smoking (5), obesity, hypertension, hyperlipidaemia (6), and certain genetic variations (7, 8). Drusen and basal linear deposits (BLinD) are the defining features of early-stage AMD. As for late-stage AMD, typically, there are two primary classifications: geographic atrophy (GA) and neovascular AMD (nAMD) (9).

Photoreceptors, retinal pigment epithelium (RPE), and choriocapillaris displayed combined degenerative alterations in GA (9). Over time, the GA area progressively expands, resulting in progressive central vision decline (10). Neovascularization may occur in the subretinal/sub-RPE space, or choroid at any stage of AMD, leading to CNV, which is a hallmark lesion of nAMD. When these newly formed blood vessels leak or rupture, nAMD enters the exudative phase. Accumulated fluid and/or hemorrhage would lead to distorted vision (11). If left untreated, CNV area typically undergo extensive fibrosis, which further deteriorates central vision (3). Over 80% of vision loss in AMD patients is attributed to nAMD (12). VEGF serves as the primary initiator for angiogenesis in CNV (13). And the introduction of anti-VEGF biologics marks a significant advancement in AMD therapy. Since the approval of anti-VEGF therapy in 2006, the number of visually impaired patients aged 80 and above with nAMD has significantly decreased. However, due to individual variability, not all patients accepting anti-VEGF treatment can maintain good vision over time stably. GA patients, on the other hand, did not respond to this therapy due to different pathogenesis. Therefore, exploring the upstream mechanisms of AMD and searching for available therapeutic targets remain urgent.

In our study, transcriptome sequencing of RPE-choroid-sclera complex (eye cup) from old mice as well as laser-induced CNV mice suggested that activated innate inflammatory response is a common mechanism of neovascularization and systemic aging, which was validated in subsequent experiments. Integrative bioinformatics analysis identified PU.1/SPI1 as a core transcription factor (TF) crucial for the development of both CNV and age-related retinal degeneration. The significant rescue in CNV model by *Spi1* interference has also highlighted its potential as an innovative target for treating AMD.

## Methods

### Animals and ethics statement

We obtained 2-month-old male C57BL/6J mice from GemPharmatech and housed them in the animal facility at Fudan University (Shanghai, China). The old C57BL/6J mice (≥18-month-old) were all provided by the research group of Professor Shi-qing Cai from the Center for Excellence in Brain Science and Intelligence Technology, Institute of Neuroscience, Chinese Academy of Sciences. The mice were provided with free access to water and solid diets, and they were maintained under standard housing conditions. Specific experiments indicated the ages and quantities of animals used, detailed either in the figure legends or the methods section. The Animal Care and Use Committee of Fudan University reviewed and approved all animal protocols, ensuring compliance with relevant regulatory standards.

### Electroretinography

The retinal function of mice was examined through electroretinography (ERG). ERG assessments were conducted following an overnight dark adaptation. Mice were situated on a heating pad throughout the experiment to ensure their body temperature. All procedures were conducted under red light. ERG was recorded in both eyes by placing electrodes on the corneas, a reference electrode was positioned on the cheek, and a neutral electrode was inserted in the tail. Mice were positioned beneath the ERG dome, and scotopic ERGs were recorded at various light intensities (0.01, 0.1, 1, 3, 10 cd.s/m2). The amplitudes of the a-wave and b-wave were analyzed using Diagnosys version 6.63 software.

### OCT

OCT examinations were conducted utilizing ISOCT (Optoprobe Science Ltd). The horizontal scan image was analyzed with the optic nerve head as the center. We used the built-in software to measure total retinal thickness at distances of 0.2, 0.4, 0.6, 0.8, and 1.0 mm from the optic nerve head (nasally and temporally).

### RNA-Seq and bioinformatics analysis

We conducted RNA-seq analysis at Majorbio Bio-pharm Biotechnology Co., Ltd. (Shanghai, China). TRIzol was utilized to extract total RNA from the eye cup. The cDNA library was prepared by 1μg of total RNA. Differentially expressed genes (DEGs) between two distinct groups were identified based on the transcripts per million reads (TPM) method. The datasets PRJNA1102502 and PRJNA1102537 in this study are available on NCBI. DEG selection criteria included |log_2_(FC)| > 1.0 and FDR < 0.05. Gene Ontology (GO) and Kyoto Encyclopedia of Genes and Genomes (KEGG) were conducted utilizing Metascape database (https://metascape.org/). The heatmap was generated on https://www.bioinformatics.com.cn. In addition, Venn’s diagram was made on https://bioinfogp.cnb.csic.es/tools/venny/index.html. We performed PPI network analysis among proteins corresponding to DEGs via STRING database (http://string-db.org). We determined key proteins in the interaction network based on betweenness centrality, and the PPI network was established through Cytoscape software (v3.8.2).

### Laser-induced CNV

Mice were given anesthesia through intraperitoneal injection of 1.25% tribromoethanol (0.3 mL/20 g) and their pupils were dilated using tropicamide. Approximately 2 minutes later, both eyes were topically treated with carbomer gel, and a small circular glass slide was placed at the center of the cornea. Using a laser photocoagulator with a wavelength of 532nm (VITRA, France), 4 or 6 laser burns were induced at 2 PD equal distances from the optic disc (4 burns for immunofluorescence experiments and 6 burns for sequencing and molecular experiments). The laser parameters were set at a power of 110mW and duration of 140ms. Successful disruption of the Bruch’s membrane was indicated by the generation of white bubbles upon photocoagulation. The laser burns were positioned to avoid retinal blood vessels. If the laser did not disrupt the Bruch’s membrane or caused retinal hemorrhage, it was excluded from analysis.

### Intravitreal injection

Intravitreal injections of AAV NC and sh*Spi1* (Obio Technology, Shanghai) were performed in the left and right eyes of 2-month-old C57BL/6J mice following pupil dilation respectively. Three weeks later, CNV model was conducted.

### Immunofluorescence

Following euthanasia of the mice, the eye globes were removed and immersed in paraformaldehyde for 30 minutes. The eye cup was dissected to remove the neuroretina and incubated in PBS containing 5% BSA and 0.5% Triton X-100 for 1 hour. Primary antibodies were applied for overnight incubation thereafter. FITC coagulated Isolectin B4 (1:100, Sigma) was used to examine blood vessels, while Iba1 (1:500, ab178846, Abcam) was utilized to identify mononuclear phagocytes. Following multiple rinses with PBS, the eye cup was then exposed to a secondary Cy3-conjugated goat anti-rabbit antibody for a duration of 1 hour. After that, the tissue was placed on a glass slide and photographed with Olympus FV3000 confocal microscope. The Z-stacks were combined into a single image for examination.

### BMDM culture

Macrophages derived from bone marrow of mice of various ages were isolated using established methods, then cultured in DMEM including 10% FBS, 1% penicillin/streptomycin, and 10ng/ml M-CSF (PeproTech). Enriched bone marrow–derived macrophages (BMDMs) can be collected for experiments after 7 days of cell culture. For *Spi* knockdown, BMDMs were infected with NC or sh*Spi1* lentivirus (Obio Technology, Shanghai) for 48 hours and harvested after 24-hour LPS stimulation.

### ELISA

We utilized ELISA kits from Multisciences Biotech to assess the protein concentrations of IL-6, IL-1β, and TNF-α in both cell culture supernatant and eye cup samples, according to the guidelines provided by the manufacturers.

### qRT-PCR

Tissues and cells were processed to extract RNA with EZBioscience kits, followed by cDNA transcription with the Color Reverse Transcription Kit from EZBioscience, according to the guidelines provided by the manufacturers. Roche LightCycler 480 II system (Roche, Switzerland) and SYBR Green (EZBioscience) were utilized to perform quantitative real-time PCR (qRT-PCR). For PCR, 2μl of cDNA was included in the 10μl overall volume as the template. The temperature cycling parameters consisted of heating to 95°C for 5 minutes, then undergoing 40 cycles of heating to 95°C for 10 seconds followed by cooling to 60°C for 30 seconds. The primer efficiency was confirmed. Following the reactions, the raw data were exported and analyzed by the 2^−△△CT^ method. The primers used are listed as follows. *Il-6*: 5’-TAGTCCTTCCTACCCCAATTTCC-3’ and 5’-TTGGTCCTTAGCCACTCCTTC-3’; *Il-1β*: 5’-GCAACTGTTCCTGAACTCAACT-3’ and 5’-ATCTTTTGGGGTCCGTCAACT-3’; *Tnf-α*: 5’-CCCTCACACTCAGATCATCTTCT-3’ and 5’-GCTACGACGTGGGCTACAG-3’; *Spi1*: 5’-ATGTTACAGGCGTGCAAAATGG-3’ and 5’-TGATCGCTATGGCTTTCTCCA-3’; *Actb*: 5’-GGCTGTATTCCCCTCCATCG-3’ and 5’-CCAGTTGGTAACAATGCCATGT-3’.

### Flow cytometry

After washing single-cell suspensions of BMDM or eye cups, they were incubated on ice for 30 minutes with specific fluorescent antibodies (PE-anti-F4/80, Biolegend, 123110; FITC-anti-CD11b, Biolegend, 101206; and APC-anti-CD11c, MULTI Sciences, AM011C05–250; or Pacific Blue-anti-CD86, Biolegend, 105123) in the dark. Samples were examined using a FACS Vantage flow cytometer from BD Biosciences, and FlowJo software was utilized to process the data.

### Statistical analysis

Analysis and figure generation were performed using GraphPad Prism 9 software. Mean ± SEM. values are depicted on the graphs. Statistical comparisons between two groups were conducted using a Student’s t-test with unequal variance assumed, while ERG and OCT data were analyzed using two-way ANOVA. *P* < 0.05 was considered statistically significant.

## Results

### Degenerative phenotypes and transcriptome changes in eyes of old mice

**Figure 1:**
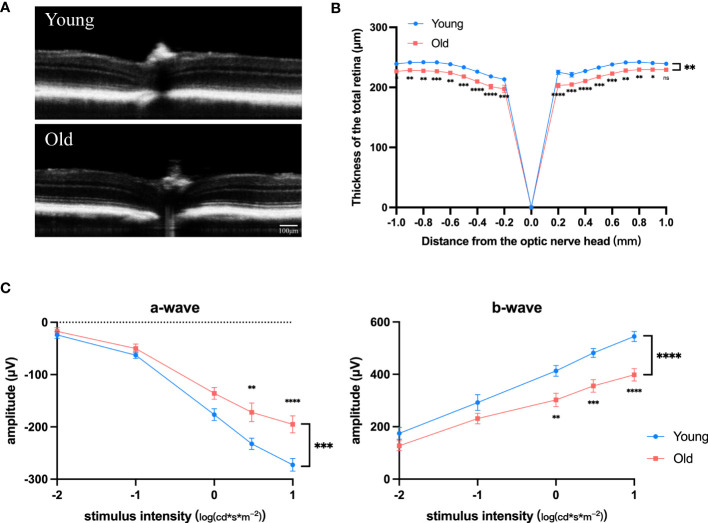
Degenerative changes in the retina of old mice. **(A)** Representative OCT images of old (18-month) and young (3-month) mice. **(B)** Total retinal thickness at varying distances (–1.0 mm to +1.0 mm) from the optic disk center (0 μm) in increments of 0.2 mm for both old and young mice, n =7. **(C, D)** Amplitudes of the a-waves **(C)** and b-waves **(D)** at different intensities in old (18-month) and young (3-month) mice, n=8. *P<0.05, **P<0.01, ***P<0.001, ****P<0.0001. All data were demonstrated as mean ± SEM.

**Figure 2:**
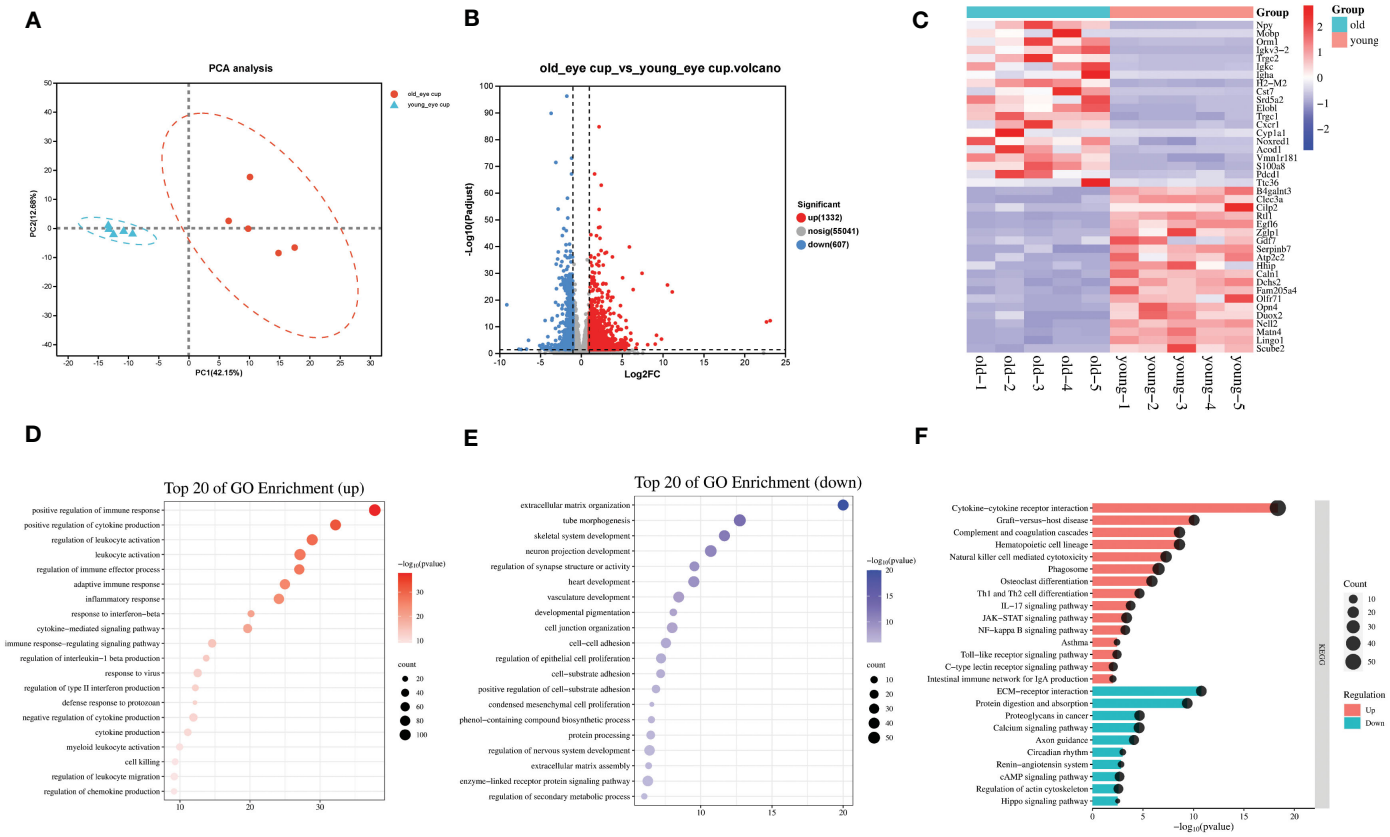
Transcriptomic analysis of the eye cups from old mice. **(A)** Principal component analysis of old (30-month) and young (3-month) eye cup, n=5. Old eye cups are marked by red and young eye cup are marked by blue. **(B)** Plot displaying DEGs in a volcano format. **(C)** Heatmap illustrating the top 20 DEGs identified between old and young eye cup. **(D, E)** Top 20 enriched GO terms among upregulated **(D)** and downregulated **(E)** DEGs in old eye cup. **(F)** Top 15 upregulated and 10 downregulated KEGG pathways in old eye cup.

As mice age, the retina experiences degenerative alterations, including a reduction in total retinal thickness (**Figures 1A, B**) and decrease of both the a and b wave amplitude in electroretinogram (**Figures 1C, D**). To investigate the underlying causes of these changes, we conducted transcriptome sequencing on the eye cups of 30-month-old elderly mice (n=5) and 3-month-old young controls (n=5). Principal component analysis (PCA) indicated that samples separated based on their experimental category (**Figure 2A**). We found 1,939 genes that were differentially expressed in old eye cup, including 1,332 upregulated and 607 downregulated genes (**Figure 2B**). A heatmap was generated for the top 20 genes that showed increased and decreased expression levels (**Figure 2C**). GO and KEGG analysis was conducted for the DEGs to illustrate functional enrichment. Top 20 upregulated GO enrichments are shown in **Figures 2D**, including positive regulation of immune/inflammation response, cytokine production, leukocyte activation, and immune effector process. These findings align with previous research suggesting the significant involvement of the immune response in eye aging. Among the top 20 downregulated GO enrichment (**Figure 2E**), extracellular matrix (ECM), neuron projection development, regulation of synapse structure or activity, vasculature development and cell junction organization were enriched. **Figure 2F** demonstrated that the KEGG analysis of increased DEGs in the old eye cup was predominantly enriched in cytokine-cytokine receptor interaction, complement and coagulation cascades, phagosome, NK cell mediated cytotoxicity, Th1 and Th2 cell differentiation, IL17, JAK-STAT and NF-κB signaling pathway. The main pathways involving down-regulated DEGs included ECM–receptor interaction, calcium signal pathway, proteoglycans in cancer, as well as protein digestion and absorption (**Figure 2F**).

### Transcripts and biological pathways altered in CNV eye cup

**Figure 3:**
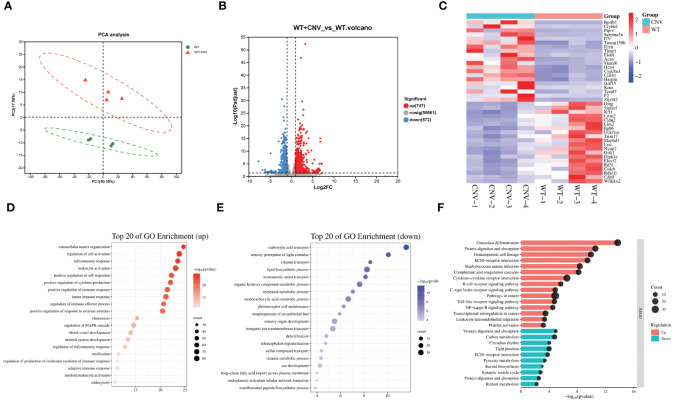
Transcriptomic analysis of the CNV eye cup. **(A)** Principal component analysis of CNV and control eye cup, n=4. Control eye cups are marked by green and CNV eye cups are marked by red. **(B)** Plot displaying DEGs in a volcano format. **(C)** Heatmap depicting the top 20 DEGs between CNV and control eye cup. **(D, E)** The top 20 enriched GO terms among upregulated **(D)** and downregulated **(E)** DEGs in CNV group. **(F)** Top 15 upregulated and 10 downregulated KEGG pathways in CNV group.

To investigate the pathogenesis of CNV, the other subtype of AMD, we used laser photocoagulation to construct a mouse model of CNV and performed transcriptome sequencing on eye cups from CNV mice (n=4) and age-matched control mice (n=4). We found that 1,319 genes were differentially expressed in eye cups with CNV, of which 747 were upregulated and 572 were downregulated (**Figures 3A, B**). A heatmap format displayed the top 20 genes that were upregulated and downregulated (**Figure 3C**). **Figures 3D, E** displayed top 20 and top 15 GO enrichments of DEGs. We found that extracellular matrix organization, leukocyte activation, inflammation/immune response and positive regulation of cytokine and chemotaxis production were enriched among the upregulated genes. Conversely, the downregulated DEGs were found to be predominantly associated with carboxylic acid transport, sensory perception of light stimulus and vitamin transport. As shown in **Figure 3F**, the KEGG indicated that upregulated DEGs were predominantly concentrated in complement and coagulation cascades, cytokine-cytokine receptor interaction, NF-κB and toll-like receptor signaling pathway. The main pathways involving downregulated genes in CNV group included the vitamin digestion and absorption, carbon metabolism, tight junction, and retinol metabolism. Notably, ECM receptor interactions were enriched among both upregulated and downregulated genes, suggesting that genes within the same term could also participate in CNV development in diverse ways.

### Common regulators involved in pathology of both aging and CNV

**Figure 4:**
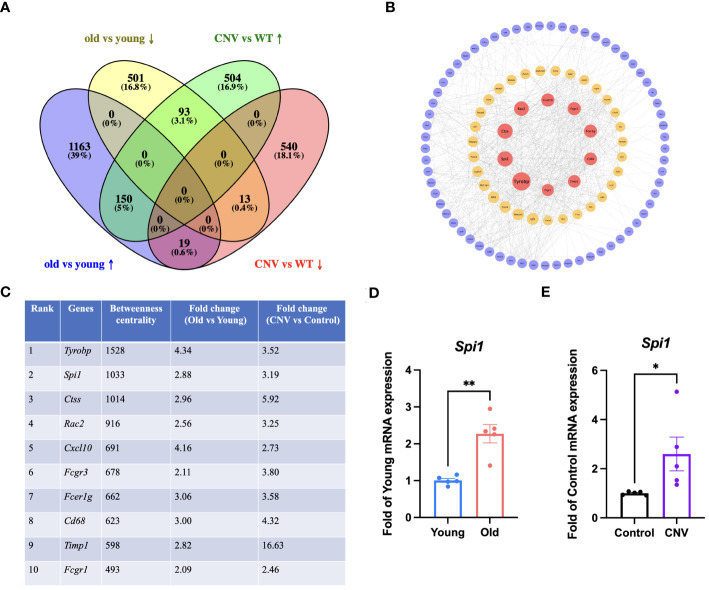
Integrative analysis of the old and CNV transcriptomics. **(A)** Venn diagram for overlapped DEGs in the old and CNV transcriptomes. **(B)** PPI network analysis of the proteins corresponding to the 150 co-upregulated DEGs in old and CNV eyes. Red: high betweenness centrality, yellow: moderate betweenness centrality, purple: low betweenness centrality. **(C)** Top 10 DEGs with highest betweenness centrality and their fold change in old and CNV transcriptomes. **(D)** mRNA expression of *Spi1* in old (30-month) and young (3-month) eye cups by qRT-PCR, n=5. **(E)** mRNA expression of *Spi1* in CNV and control eye cups by qRT-PCR, n=5. *P<0.05, **P<0.01. All data were demonstrated as mean ± SEM.

Considering that CNV and age-related retinal degeneration are two representative aspects of pathologic changes of AMD, we conducted integrative analysis on the 2 sets of transcriptomic data described above to reveal their common pathogenesis. We identified a total of 275 overlapped DEGs (**Figure 4A**). Out of the total, 150 genes showed simultaneous upregulation, while 13 genes exhibited simultaneous downregulation in both old and CNV mice. We conducted a PPI network analysis on the proteins corresponding to the 150 co-upregulated DEGs in both old eyes and CNV eyes to determine the main pathological factors contributing to AMD at the molecular level (**Figure 4B**). The calculation of betweenness centrality for each node identified the key proteins that act as communication bottlenecks in the network. Node size reflects the degree of connections while node color represents the betweenness centrality. Red markers indicate the core proteins with the highest values in the figure. Generally, high betweenness centrality implies important biological roles and could be a hint of potential therapeutic targets. Top 10 genes with highest betweenness centrality are *Tyrobp, Spi1, Ctss, Rac2, Cxcl10, Fcgr3, Fcer1g, Cd68, Timp1* and *Fcgr1*. The betweenness centrality and fold change of these genes are listed in **Figure 4C**. Subsequently, the elevated mRNA expression level of a TF, *Spi1*, was verified in eyes from old mice and CNV mice compared with their respective controls (**Figures 4D, E**).

### Systemic pro-inflammatory status in old mice

**Figure 5:**
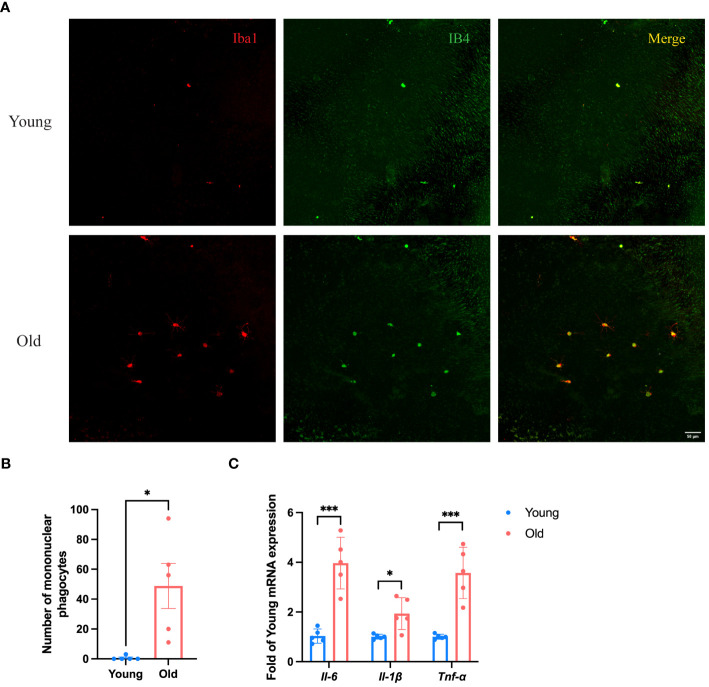
Eye cup from old mice exhibited a pro-inflammatory state. **(A)** Representative confocal images of IB4 and Iba1 stained activated MNPs of choroid flat mounts in old (24-month) and young (3-month) mice. **(B)** Numbers of IB4^+^Iba1^+^ MNPs in subretinal space of young and old mice, n=5. **(C)** Pro-inflammatory factors in old (30-month) and young (3-month) eye cup quantified by qRT-PCR, n=5. *P<0.05, ***P<0.001. All data were demonstrated as mean ± SEM.

**Figure 6:**
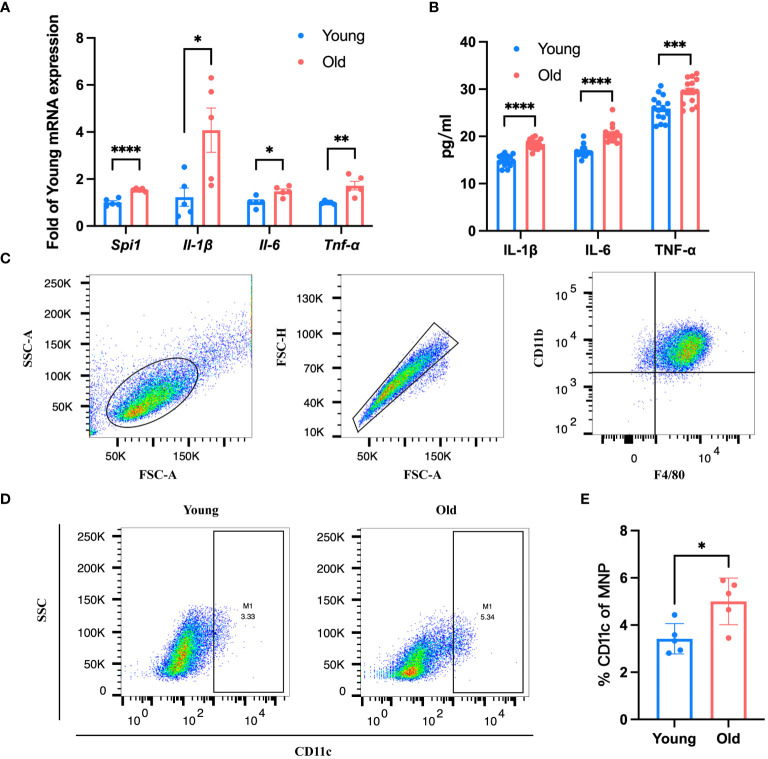
BMDMs from old mice presented a pro-inflammatory state. **(A, B)** *Spi1* and pro-inflammatory factors in old (21-month) and young (3-month) BMDMs quantified by qRT-PCR **(A)** and Elisa **(B)**, n=5. **(C)** Flow cytometry analysis strategy of BMDMs from old and young mice. **(D)** Representative flow plots of M1 macrophage in BMDMs of old and young mice. **(E)** Quantification of the proportion of M1 macrophages in BMDMs from old and young mice, n=5. *P<0.05, **P<0.01, ***P<0.001, ****P<0.0001. All data were demonstrated as mean ± SEM.

According to the results of scRNA-seq in the database, *Spi1* is highly expressed in macrophages, positively correlated to innate immune responses. We performed co-staining of IB4 and Iba1 on choroid flat mounts of young and old mice to label activated mononuclear phagocytes (MNPs) dislocated in the subretinal space and quantitatively analyze them (**Figures 5A, B**). Unsurprisingly, young mice had almost no or very few activated MNPs in the subretinal space, whereas old mice exhibited an elevation in the number. qRT-PCR analysis of pro-inflammatory factors of eye cup, including *Il-6, Il-1β*, and *Tnf-α* revealed a significant elevation in old group (**Figure 5C**). BMDMs were cultured from both young and old mice for *in vitro* experiments. And we conducted additional investigations on the expression differences in *Spi1* and pro-inflammatory factors at cellular level. We confirmed that *Spi1* and pro-inflammatory factors were elevated in BMDMs from old mice (**Figure 6A**). Additionally, the concentrations of inflammatory proteins in the cell supernatant were notably higher in the older group (**Figure 6B**). Flow cytometry analysis was conducted on BMDMs utilizing F4/80 and CD11b markers to distinguish macrophages, as well as the CD11c marker to identify M1 macrophages (**Figures 6C, D**). As shown in **Figure 6E**, the study found that the percentage of M1 macrophages in the elderly group was 1.5 times greater than the young mice,.

### Pro-inflammatory alternation and M1 polarization were shared in CNV eye cup

**Figure 7:**
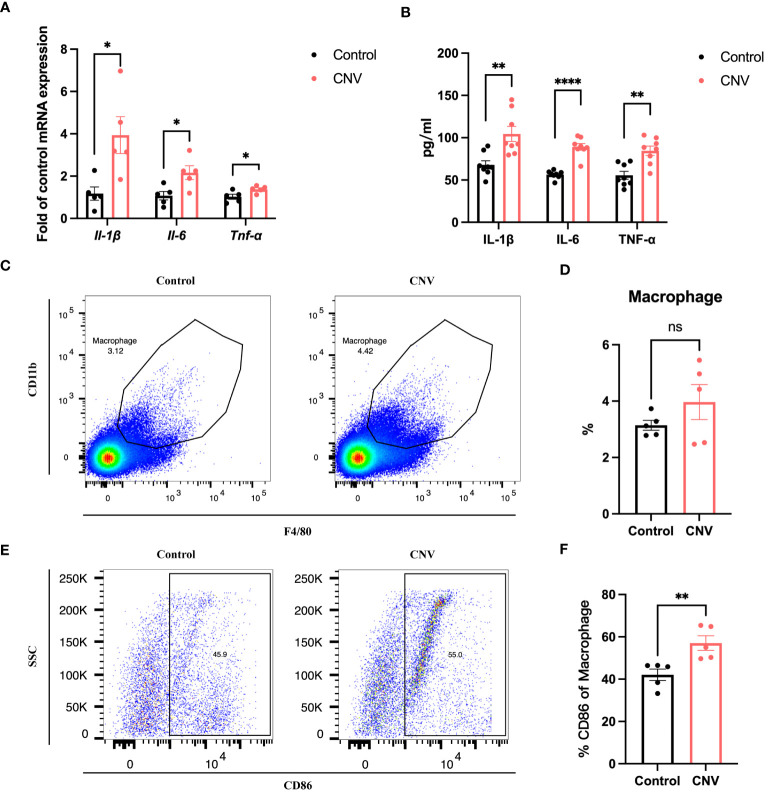
Eye cup from CNV mice displayed a pro-inflammatory state. **(A, B)** Pro-inflammatory factors in CNV and control eye cup quantified by qRT-PCR **(A)** and Elisa **(B)**, n=5. **(C)** Representative flow plots of macrophages in eye cup of CNV and control mice. **(D)** The proportion of macrophages in CNV and control group, n=5. **(E)** Representative flow plots of M1 macrophages in eye cup of CNV and control mice. **(F)** Quantification of the proportion of M1 macrophages in CNV and control mice, n=5. *P<0.05, **P<0.01, ****P<0.0001, ns, not significant. All data were demonstrated as mean ± SEM.

To investigate whether SPI1 also affects the inflammatory status of the eye cup in CNV mice, qRT-PCR and ELISA analysis were performed, revealing an elevation in pro-inflammatory factors (**Figures 7A, B**). Flow cytometry analysis of eye cup was conducted on control and CNV mice, showing an increased trend in the proportion of F4/80^+^CD11b^+^ macrophages (**Figures 7C, D**) as well as a higher proportion of M1 macrophages (**Figures 7E, F**) in eye cup from CNV mice.

### Knockdown of *Spi1* inhibited M1 polarization of macrophages and alleviated CNV

**Figure 8:**
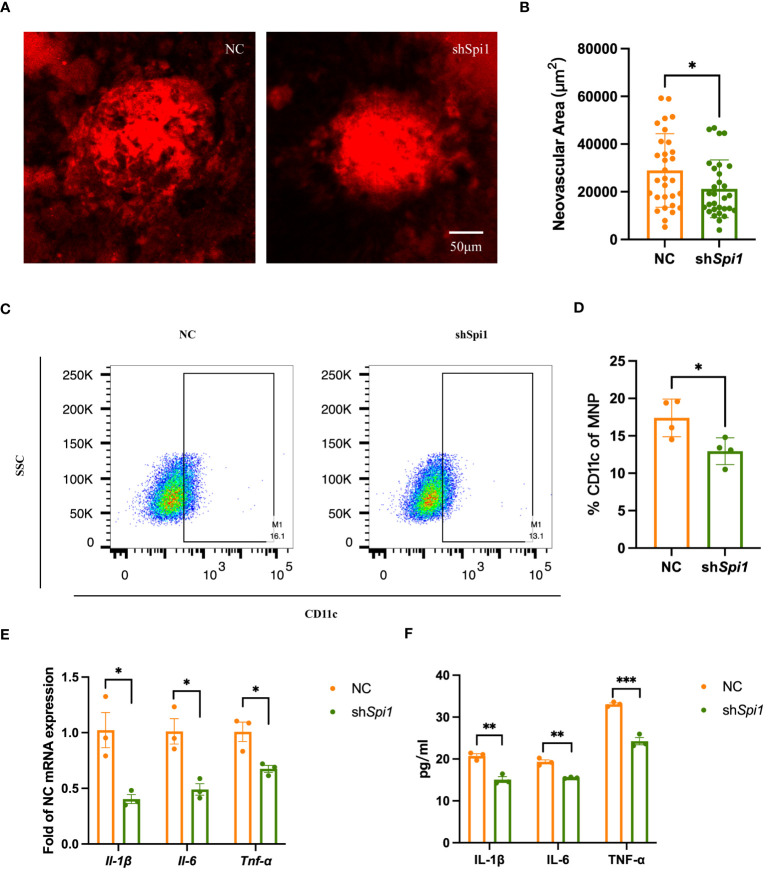
*Spi1* knockdown alleviated CNV by inhibiting M1 polarization. **(A)** Representative confocal images of IB4-stained neovascularization area in NC and sh*Spi1* eye cups of CNV mice. **(B)** Quantification of the IB4-labeled CNV area in NC and shSpi1 groups (n=8). **(C)** Representative flow plots of M1 macrophages in NC and sh*Spi1* BMDMs. **(D)** The proportion of M1 macrophages in NC and shSpi1 BMDMs, (n=3). **(E, F)** Pro-inflammatory factors in NC and shSpi1 BMDMs quantified by qRT-PCR **(E)** and Elisa **(F)**, (n=3). *P<0.05, **P<0.01, ***P<0.001. All data were demonstrated as mean ± SEM.

To further validate the role of SPI1 in inflammatory response and AMD, we assessed the neovascular process in CNV mice with *Spi1* knockdown. 2-month-old mice were intravitreally injected with AAV carrying either NC or *Spi1* knockdown constructs in their left and right eyes, respectively. After three weeks, CNV modeling was performed, and choroid flat mounts were obtained one week later for IB4 staining to label the area of neovascularization. It was observed that *Spi1* knockdown significantly reduced laser-induced neovascularization area (**Figures 8A, B**). *In vitro* cell experiments were also performed on extracted BMDMs with *Spi1*-knockdown lentivirus. After 48 hours, the cells were exposed to LPS for an additional 24 hours before being harvested to assess polarization. It was found that *Spi1* knockdown markedly inhibited macrophage polarization towards the M1 phenotype (**Figures 8C, D**), leading to a notable decrease in pro-inflammatory factors (**Figures 8E, F**).

## Discussion

Inflammation activation is similar in age-related retinal disorders and CNV, which are two major pathological changes of AMD. We performed integrative analysis of old and CNV transcriptomic features to confirm the broad proinflammatory state and identify a core transcription factor, SPI1. Our findings revealed a significant upregulation of SPI1 expression in both old mice and CNV models, suggesting that it is closely related to aging and neovascularization. Through gene knockdown experiments targeting *Spi1* in BMDM and CNV mice, we observed a significant inhibition of macrophage M1 polarization and a reduction in neovascularization, suggesting it as a potential therapeutic target for AMD.

Our study of the old mice provided insights into both the phenotypic and transcriptomic changes associated with aging in the eye. The process of aging is intricate, impacting different facets of the structure and function of the retina, ultimately raising the likelihood of developing age-related retinal conditions. The phenotypic findings we obtained aligned with prior research, revealing significant alterations in the aging mouse retina such as reduced thickness of retinal layers and diminished a and b wave amplitudes in electroretinogram recordings (14, 15). Furthermore, these alterations are accordance with either functional decline or structural abnormalities often seen in age-related retinal disorders, such as AMD (15), providing a basis for understanding the development of retinal pathologies with natural aging. Complementing the phenotypic changes, our transcriptomic analysis revealed significant alterations in gene expression profiles in the eye cup from old mice. It was discovered that osteocytes could promote fat formation and constrain bone formation of bone marrow mesenchymal stem cells through the secretion of neuropeptide Y (NPY). and the expression of NPY rises with aging and in cases of osteoporosis (16). We found that NPY increased to the highest degree in old mice, which may lead to the occurrence of degenerative diseases due to the disturbance of retinal metabolic regulation. Previous studies employed senescence-accelerated mouse prone 8 (SAMP8) as an experimental model to investigate neurodegenerative diseases. It was found that MOBP expression is downregulated in SAMP8 mice, potentially playing a role in age-related brain diseases (17). However, our sequencing revealed that MOBP is significantly upregulated in the eye cup of old mice. This could be due to tissue differences as well as differences in normal aging and aging induced by genetic editing. Among the remaining significantly upregulated genes, some are functionally related to aging immune regulation, such as *Trgc*, *Pdcd1*, *S100a8*, and others. Administering PD-1 antibody to aging mice or non-alcoholic steatohepatitis (NASH) mice lead to the reduction on the number of p16^+^ cells as well as the activated CD8^+^ T cell-dependent PD-L1^+^ population *in vivo*, ameliorating various aging-related phenotypes (18). S100A8-positive microglia are upregulated in tau disease mouse models and SAMP8 mice, and S100A8^+^ microglia were found in brain biopsies from individuals with AD and elderly patients with no reported pathology, which may be associated with cognitive dysfunction (19). GO and KEGG reflected a wide range of biological processes and molecular pathways implicated in ocular aging and AMD. Aligning with previous studies, inflammation response and ECM remodeling turned to be the most dysregulated pathways, highlighting their irreplaceable role (20, 21). Under typical circumstances, the blood-retinal barrier (BRB) collaborates with immune effectors to uphold retinal balance. With aging, both the retinal vascular endothelial cells and RPE cells experience age-related functional impairments, including the weakening of physical barriers such as the inner and outer BRBs (22, 23). As for the immune effectors of the retina, including microglia and the complement system, they also experience notable alterations with age (24, 25). On one side, senescent microglia show reduced capacity in immune surveillance and synaptic maintenance, bringing about the deposition of defective neurons and fragments. Conversely, microglia and the complement system exhibit an increased pro-inflammatory response to damaged neurons and debris, contributing to immune pathology. These aging-related alterations would violate intraocular microenvironment and make it more prone to conditions like AMD, diabetic retinopathy, and glaucoma (26, 27).

In addition to the degenerative changes occurring with aging, CNV, which is identified by the abnormal expansion of blood vessels from choroid into retina, is another major subtype of AMD that leads to over 80% of vision impairment in affected individuals. We conducted transcriptome sequencing of eye cups from a mouse model with laser induced CNV to discover important genes and pathways related to CNV. Significant changes in gene expression pattern were revealed, encompassing multiple signaling pathways mainly associated with inflammation, tight junction, and ECM remodeling (11, 26, 28). Among them, changes in various cytokines were the most striking, including IL-1β, IL-2, and IL-6. It has been reported that they not only participate in neovascularization and extensive fibrosis but are also closely associated with dry AMD. The effectiveness of IL-1Ra in reducing CNV is attributed to blocking IL-1α and IL-1β (29–31). In our study, we found a significant upregulation of *Il1rn *in CNV mice. This may be due to a protective response mounted by the organism in response to the disease state. However, as the disease progresses and exceeds the body’s self-regulatory capacity, this response may become insufficient. As reported in the literature, in the early stages of CNV, local administration of IL-1ra significantly inhibits CNV development. However, treatment initiated one week after CNV induction only transiently suppresses the vascular response (32). IL-2 is pivotal in fibrosis of macular degeneration via participation in the migration of RPE cells, synthesis of ECM, and expression of TGF-β2 (33). IL-6 holds importance in promoting subretinal fibrosis and is involved in the repair of damaged organs (34). Serum IL-6 levels are associated with the course of GA (35). Additionally, studies have shown that the activation of STAT3 through IL-6 receptor contributes to the development of CNV, with higher levels of IL-6 in the aqueous humor of AMD patients correlating with increased CNV size and activity (34–36). In the DEGs of CNV mice, a significant downregulation of several genes related to neuronal function is observed. OMG, LRTM2, GRIK1, and CDH9 are predominantly expressed in the nervous system, participating in neuronal and synaptic functions, has a significant impact on the occurrence of various neurological disorders. LRAT and RDH10 play crucial roles in the visual process, participating in the vitamin A metabolic pathway, which is essential for normal visual system function. Mutations in LRAT and RDH10 genes are essential for the occurrence of retinal degenerative diseases.

The identification of shared KEGG pathways involved in inflammation and ECM remodeling altered in eye cups from both old mice and the CNV model suggests common pathogenic mechanisms underlying. Considering that aging-related retinal degeneration and CNV progression are two classic branches of AMD lesions, we performed integrative analysis of these two set of transcriptomics in search of potential upstream therapeutic targets for AMD. It was revealed that 150 genes were significantly co-upregulated. Subsequently, PPI analysis was conducted and the 150 DEGs were sorted according to their betweenness centrality. While the top 10 core DEGs have distinct functions, they share some common characteristics. Overall, these genes collectively contribute to immune responses, inflammation, and immune cell functions, coinciding the common pathology shared by two subtypes of AMD. Their coordinated actions are crucial for maintaining immune homeostasis and defending against infectious agents as well as other challenges to the immune system. It is noteworthy that Timp1 is drastically upregulated in the CNV group to a much greater extent than the other nine genes. TIMP1 exerts its function by forming a complex with metalloproteinases, inhibiting their activity, and thereby maintaining the stability of the ECM and the normal structure of tissues. It has significant effects on cell migration, tumor development, and angiogenesis. Epoxomicin may lead to an imbalance between MMP-2 and TIMP-1 in ARPE-19 cells, resulting in the occurrence of retinal fibrosis (37). Since in most cases, transcription factors (TFs) are always the key regulators of gene expression and cellular functions, and their dysregulation has been implicated in various pathological conditions including neovascular diseases (38, 39), we focused on the only TF among the top 10 pivotal DEGs, SPI1, for further study.

SPI1, also referred to as PU.1, is mainly found in hematopoietic cells and lymphocytes, and is recognized as a key controller of myeloid cell development and macrophage terminal differentiation (40). Prior investigations indicated a positive association between SPI1 expression and the progression of AD as well as Aβ deposition. To be specific, SPI1 binding motifs were found to be enriched in the promoters of genes associated with disease, and SPI1 was observed to promote disease advancement by transcriptionally regulating these downstream genes (41). Additionally, SPI1 can modulate liver metabolic function by influencing macrophage polarization, making it a promising therapeutic target for type 2 diabetes (T2D) and NAFLD/NASH (42). To date, the majority of research concerning SPI1 in the retina has predominantly centered on ocular inflammation, including conditions such as uveitis, optic nerve injury, and photoreceptor malnutrition (43–45). Significant inhibition of IFN-γ and IL-2 production stimulated by IRBP was observed with SPI1 knockdown, alleviating autoimmune uveoretinitis (44). What’s more, SPI1 can synergistically regulate the activation of microglia with IRF8 and is involved in neurodegeneration caused by traumatic injury (45). Sun et al. also discovered that blocking SPI1 could reverse the damage to RPE caused by Aβ by decreasing the production of ROS and alleviating mitochondrial dysfunction (46). Given the active involvement of macrophages in the progress of CNV and aging which was confirmed in our study, we were curious whether SPI1 also contributed significantly to the onset of AMD through regulating mononuclear macrophage system, which was finally proved in subsequent experiments. As reported in the literature and observed in our experiments, macrophages are essential in the development of both CNV and age-related retinal degenerative alterations, particularly the M1 phenotype being associated with pro-inflammatory responses and angiogenesis promotion (47, 48). This is to be expected, as prolonged intraocular damage would lead to the overactivation of microglia, specific macrophages resident in the eye, secretion of multiple pro-inflammatory and cytotoxic factors as well. They create a pro-inflammatory environment and in turn further recruit more retinal microglia as well as exogenous monocytes. Sustained inflammatory damage is central to the initiation of GA, providing a breeding ground for angiogenesis (49, 50). Our findings further highlighted the involvement of SPI1 in macrophage polarization and its impact on disease progression. The suppression of Spi1 expression led to significant reduction in CNV as well as alleviation of M1 polarization in BMDMs. This suggests that by attenuating the M1 polarization of macrophages, SPI1 inhibition may help modulate the inflammatory microenvironment and reduce the neovascularization process, potentially offering a new treatment approach for AMD.

Although our study focused on SPI1 and its impact on CNV, it is important to acknowledge that CNV pathogenesis is a complex process involving multiple factors and cellular interactions. Other transcription factors, signaling pathways, and cellular components may also contribute to CNV development and warrant further investigation. In addition, we only performed *Spi1* knockdown in BMDMs to examine its regulation of the production of pro-inflammatory factors *in vitro*. Phenotypic analysis of old mice with SPI1 interference *in vivo* is helpful to further understand its role in immunosenescence.

In summary, using transcriptomic sequencing in eye cups from old and CNV mice, we demonstrated the hyperactivation of innate immune response as a shared alternation of biological process, and identified a core TF SPI1. Subsequently, *Spi1* was validated to be upregulated not only in the eye cup from two groups but also senescent BMDMs, accompanied by polarization of macrophages towards the M1 phenotype. Knocking down *Spi1* resulted in the inhibition of M1 polarization of macrophages, relieved the pro-inflammatory microenvironment and alleviated CNV. That’s to say, SPI1 holds great potential as a new therapeutic target for AMD because of its regulatory role in the general pathologic mechanism upstream.

## Data availability statement

The datasets presented in this study can be found in online repositories. The names of the repository/repositories and accession number(s) can be found in the article/Supplementary Material.

## Ethics statement

The animal study was approved by The Animal Care and Use Committee of Fudan University. The study was conducted in accordance with the local legislation and institutional requirements.

## Author contributions

SQ: Conceptualization, Data curation, Formal analysis, Investigation, Methodology, Project administration, Software, Supervision, Validation, Visualization, Writing – original draft, Writing – review & editing. YZ: Conceptualization, Formal analysis, Investigation, Methodology, Project administration, Software, Supervision, Writing – original draft, Writing – review & editing. LK: Data curation, Formal analysis, Investigation, Methodology, Software, Supervision, Visualization, Writing – review & editing. DB: Investigation, Methodology, Validation, Visualization, Writing – review & editing. HK: Writing – review & editing, Investigation, Methodology, Software. SZ: Supervision, Validation, Writing – review & editing, Visualization. CZ: Funding acquisition, Project administration, Resources, Supervision, Validation, Writing – review & editing.
